# Use of metabolomics for the chemotaxonomy of legume-associated *Ascochyta* and allied genera

**DOI:** 10.1038/srep20192

**Published:** 2016-02-05

**Authors:** Wonyong Kim, Tobin L. Peever, Jeong-Jin Park, Chung-Min Park, David R. Gang, Ming Xian, Jenny A. Davidson, Alessandro Infantino, Walter J. Kaiser, Weidong Chen

**Affiliations:** 1 Department of Plant Pathology, Washington State University, Pullman, WA 99164, USA; 2 Institute of Biological Chemistry, Washington State University, Pullman, WA 99164, USA; 3 Department of Chemistry, Washington State University, Pullman, WA 99164, USA; 4 South Australian Research and Development Institute, Adelaide, South Australia 5001, Australia; 5 Consiglio per la Ricerca in Agricoltura e l’analisi dell’economia agraria (CREA), Centro di Ricerca per la Patologia Vegetale, Rome, 00156, Italy; 6 USDA-ARS Western Regional Plant Introduction Station, Washington State University, Pullman, WA 99164, USA; 7 USDA-ARS Grain Legume Genetics and Physiology Research Unit, Washington State University, Pullman, WA 99164, USA

## Abstract

Chemotaxonomy and the comparative analysis of metabolic features of fungi have the potential to provide valuable information relating to ecology and evolution, but have not been fully explored in fungal biology. Here, we investigated the chemical diversity of legume-associated *Ascochyta* and *Phoma* species and the possible use of a metabolomics approach using liquid chromatography-mass spectrometry for their classification. The metabolic features of 45 strains including 11 known species isolated from various legumes were extracted, and the datasets were analyzed using chemometrics methods such as principal component and hierarchical clustering analyses. We found a high degree of intra-species consistency in metabolic profiles, but inter-species diversity was high. Molecular phylogenies of the legume-associated *Ascochyta*/*Phoma* species were estimated using sequence data from three protein-coding genes and the five major chemical groups that were detected in the hierarchical clustering analysis were mapped to the phylogeny. Clusters based on similarity of metabolic features were largely congruent with the species phylogeny. These results indicated that evolutionarily distinct fungal lineages have diversified their metabolic capacities as they have evolved independently. This whole metabolomics approach may be an effective tool for chemotaxonomy of fungal taxa lacking information on their metabolic content.

Many species in the fungal genera *Ascochyta* and *Phoma* are recognized as primary or opportunistic pathogens on different plants of agricultural and economical importance, including some notorious pathogens with quarantine status^1^. Some species are known to produce mycotoxins, which could directly threaten animal and human health^2^,^3^,^4^. The genera *Ascochyta* and *Phoma* both are polyphyletic, being embedded in the order Pleosporales within the class Dothideomycetes^5^. Species of *Ascochyta* and *Phoma* share morphological and physiological features, and produce similar disease symptoms on plants^5^. In the Saccardoan system, *Ascochyta* and *Phoma* are distinguished only by conidial morphology; two-celled conidia in the former and one-celled conidia in the latter^6^. However, the two-celled conidial character appears to have evolved independently multiple times during evolution of several lineages in Pleosporales. The type of conidiogenous cells has also been used as an ultrastructural character to distinguish the two genera^6^. However, this character is not consistent with the conidial ontogeny of *Phoma* species in some instances^7^. Due to lack of reliable morphological characters to distinguish the two genera as well as a high degree of environmental variation, systematics of these genera has never been fully resolved^1^. This has resulted in unclear taxonomic placement with many species having both *Ascochyta* and *Phoma* names^5^.

The internal transcribed spacer (ITS) region of the nuclear rDNA operon has been proposed as a universal DNA barcode that can be used to circumscribe species boundaries of many fungi^8^. However, this locus is sometimes not suitable for delimitation of closely related taxa^9^. *Phoma* is one of the largest and the most complex fungal genera, with more than 3,000 infrageneric taxa described^10^. To resolve the phylogeny of *Phoma* at the species level or below, several protein-coding genes such as cytochrome *c* oxidase subunit I (*COI*), actin and *β*-tubulin, have been used^11^,^12^. Although the *COI* has been successfully applied to *Penicillium* taxonomy^13^, the locus does not exhibit taxon-specific conserved single nucleotide polymorphism (SNP) in a subset of *Phoma* taxa^11^. The actin locus showed a large number of SNPs and provided enough characters to resolve the phylogeny of *Phoma* below the species level^11^. However, interspecific variation is very high and, as a result, sequence alignments may be equivocal resulting in the loss of informative sites^11^. An additional challenge that has been observed with faster-evolving loci is topological incongruence among loci. A phylogeny estimated using sequence data of the *β*-tubulin locus was incongruent with a phylogeny estimated from other housekeeping genes among legume-associated *Ascochyta* species, possibly due to incomplete lineage sorting and/or gene flow and hybridization^12^.

A polyphasic approach integrating morphological and physiological features with DNA sequence data is necessary to classify diverse *Ascochyta* and *Phoma* spp.^5^. Chemotaxonomy is one method of biological classification based on the similarity of chemical compounds such as carbohydrates, lipids, amino acids or secondary metabolites (SM)^14^,^15^,^16^. Filamentous fungi, especially ascomycetes, are known to produce a vast array of SMs such as terpenes, polyketides, non-ribosomal peptides, as well as many other small organic compounds of mixed biosynthetic origin. Extensive genome sequencing efforts have revealed that genomic differences between fungal species are often related to the number and similarity of genes involved in SM biosynthesis like polyketide synthases and non-ribosomal peptide synthetases, which result in the production of a unique set of SMs^17^,^18^,^19^. Therefore, it is tempting to propose the use of SM profiles as a taxonomic diagnostic tool, and the chemotaxonomy is proven to be effective in *Aspergillus* and *Penicillium* classification^20^,^21^,^22^.

Several *Ascochyta* species infect cool season food legumes in a host-specific manner. *Ascochyta fabae* Speg., *A. lentis* Vassiljevsky, *A. pisi* Lib., *A. rabiei* (Pass) Labr., and *A. viciae-villosae* Ondrej are pathogens of faba bean (*Vicia faba* L.), lentil (*Lens culinaris* Medik.), pea (*Pisum sativum* L.), chickpea (*Cicer arietinum* L.), and hairy vetch (*Vicia villosa* Roth), respectively^23^,^24^,^25^. These legume-associated *Ascochyta* fungi form a monophyletic group that also includes other *Ascochyta* and *Phoma* spp. sampled from non-legume hosts^26^,^27^. Taxa infecting different legume hosts can be differentiated through phylogenetic analyses employing DNA sequences of multiple protein-coding genes with each host-specific taxon grouped into a separate, well-supported clade^26^,^27^. In addition to phylogenetic species recognition, the biological species recognition has been employed to describe these closely related species^28^,^29^. *In vitro* genetic crosses were made between strains of *A. rabiei*, *A. fabae* and *A. lentis*^28^. *Ascochyta rabiei* was able to mate neither with *A. fabae* nor *A. lentis*. In contrast, the genetic crosses between *A. fabae* and *A. lentis* strains produced sexual fruiting bodies with viable ascospores, although intrinsic postzygotic mating barriers were observed, such as nonstandard numbers of ascospores in asci, variable size of ascospores, and poor viability and growth suggesting that the three taxa represent biological species^28^. In a separate study, genetic crosses were made between *A. pisi* and *A. fabae* and between *A. lentis* and *A. viciae-villosae*^29^. In both combinations, normal asci with viable ascospores were produced and molecular markers segregated normally, indicating a lack of intrinsic mating barriers. However, the pathogenic abilities of the progenies derived from the cross between *A. pisi* and *A. fabae* were greatly impaired, indicating that genes controlling host specificity likely represent extrinsic postzygotic mating barriers acting to prevent hybrid formation and thereby maintain species integrity. These results revealed that the taxa used in these crosses were closely related yet biologically distinct^29^.

Legume-associated *Ascochyta* and *Phoma* species are well studied from a phylogenetic perspective^26^, which allows us to examine the utility of chemotaxonomy for identification and to test its concordance with previous classification based on biological and phylogenetic species concepts. To date, however, a major problem for the application of chemotaxonomy is that only a few SMs have been identified from *Ascochyta* and *Phoma* species. Metabolomics is the comprehensive analysis of the biochemical content of cells, tissues or entire organisms, usually from analysis of extracts. Liquid chromatography, coupled with time-of-flight mass spectrometry (LC-TOF-MS), is a powerful tool for metabolic profiling in many fields of biology^30^,^31^. The whole metabolomes of fungal extracts can be compared for chemotaxonomic classification when limited information is available for metabolite production of particular species of interest.

We conducted metabolic profiling of 11 described *Ascochyta* and *Phoma* spp. as well as several *Ascochyta* fungi isolated from cultivated legumes or related wild legumes that have not yet been fully characterized or described. The primary objective of this research was to assess the feasibility that *Ascochyta* and *Phoma* species could be differentiated according to their metabolic profiles and allow a test of previous species delimitations based on phylogenetic and biological species concepts. A secondary objective was to determine the evolutionary relationships of provisionally identified strains with known *Ascochyta*/*Phoma* spp. by performing multivariate chemometrics analyses with their metabolic profiles, and mapping that to a phylogenetic analysis based on DNA sequence data. A tertiary objective was to identify SMs from newly identified species such as *Phoma koolunga*. This is a newly described member of the ascochyta blight complex of field pea found in southern and western Australia^32^.

## Results

### Metabolic profiling of legume-associated *Ascochyta* and *Phoma*

To profile the metabolomes of legume-associated *Ascochyta* and *Phoma* spp., a total of 45 strains were cultivated on autoclaved oat kernels, and culture extracts were subjected to LC-MS analyses. Metabolic profiles were further processed and analyzed using multivariate chemometrics methods to assess intra-species consistency and inter-species differences in metabolite production. A preliminary study confirmed that oat kernel cultures of the tested strains yielded a diverse array of metabolites, including previously known SMs of the tested strains, such as ascochitine, solanapyrone A, and pinolidoxin produced by *A. pisi*, *A. rabiei*, and *A. pinodes*, respectively^33^,^34^,^35^.

**Figure 1:**
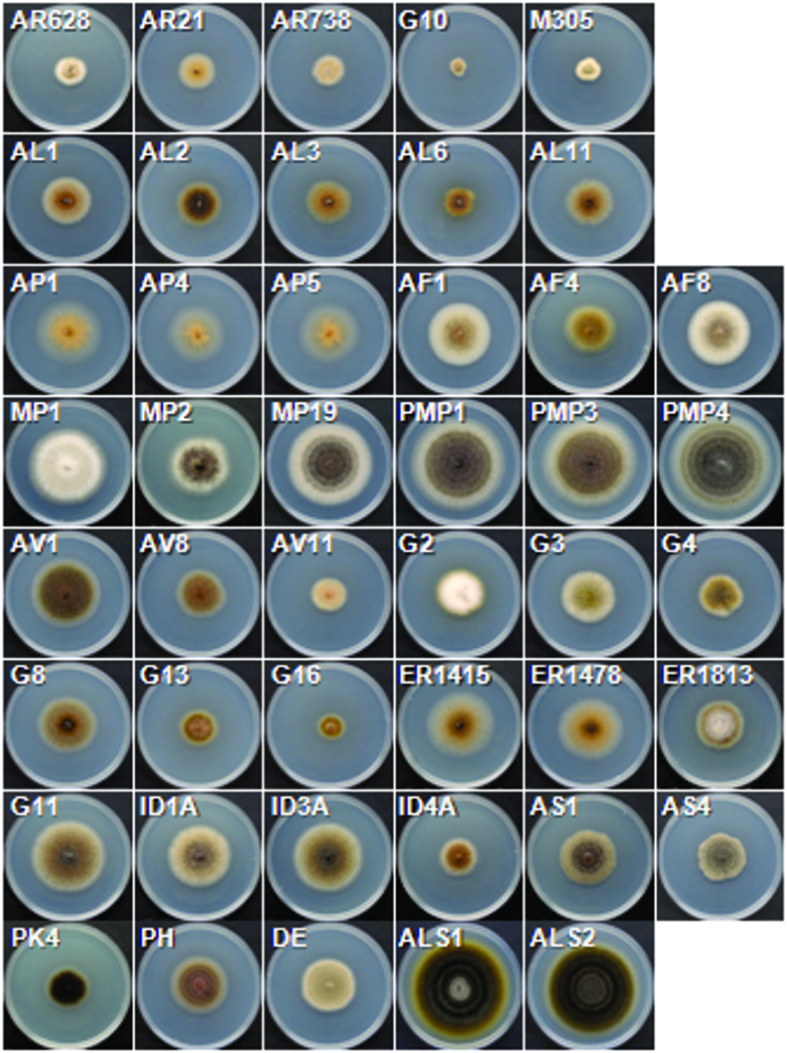
Colony morphology of 45 *Ascochyta*, *Phoma*, and *Alternaria* strains. Strains were indicated by their respective codes. Detailed information of the strains is in Supplementary Table S1. Two *Alternaria solani* strains (ALS1 and ALS2) were also included in this study as an outgroup for the *Ascochyta* and *Phoma* taxonomy. Photos were taken 1 week after incubation on PDA.

Two independent experiments were carried out for the metabolic profiling of the *Ascochyta*/*Phoma* strains. In the first experiment, we were interested in testing the hypothesis that legume-associated *Ascochyta* species can be distinguished and recognized by their metabolic profiles. We analyzed 21 fungal strains including well-described species such as *A. pinodes*, *A. pisi*, and *A. rabiei* whose teleomorphs (sexual stage) have been connected to *Didymella*^26^,^27^,^36^. In the second study, we included more diverse strains that were less well characterized but provisionally identified as *Ascochyta* species based on morphology and phylogenetic analysis. Also included were the type species of *Ascochya*, *Phoma*, and *Didymella* (*A. pisi*; *Phoma herbarum* Westend.; *Didymella exigua* (Niessl) Sacc.). The details on the strains used in the analyses are described in Supplementary Table S1, and their colony morphology is shown in Fig. 1.

### Chemoconsistency

Initially, we chose six *Ascochyta* and *Phoma* species; *A. pinodella* (strain codes: PMPs), *A. pinodes* (MPs), *A. pisi* (APs), *A. rabiei* (ARs, G10, and M305), *P. koolunga* (PK4), and *P. medicaginis* (ASs). Strains representing the same species from different geographical origins were used, when possible (see Supplementary Table S1) to assess the consistency of metabolite production across regions. Strains (ID1A, ID3A, ID4A, and G11) that were morphologically and phylogenetically similar to *Ascochyta* species^26^,^27^,^37^,^38^, were also included to see if their metabolic features are similar to or distinct from known species.

**Figure 2:**
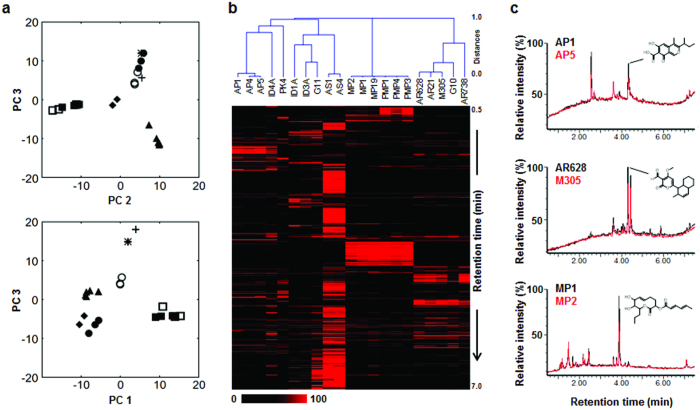
Metabolic profiles among 21 *Ascochyta* and *Phoma* strains. (**a**) PCA scores plot (upper), PC 2 versus PC 3 showing the variation in the metabolic profiles from 21 fungal strains: AP1, AP4, and AP5 (closed circles: *A. pisi*); ID4A (cross: an *Ascochyta*-like strain); PK4 (asterisk: *P. koolunga*); ID1A, ID3A, and G11 (open circles: *Ascochyta*-like strains); AS1 and AS4 (diamonds: *P. medicaginis*); MP1, MP2, and MP19 (open squares: *A. pinodes*); PMP1, PMP3, and PMP4 (closed squares: *A. pinodella*); AR628, AR21, AR738, G10, and M305 (triangles: *A. rabiei*). PLS scores plot (lower), showing the supervised separation of the strains. (**b**) Dendrogram of the 21 strains based on chemical similarity, based on UPGMA clustering method. The heatmap of respective metabolites corresponding to each strain is presented. (**c**) Overlaid total ion chromatograms of *A. pisi* strains in upper panel, *A. rabiei* strains in middle panel, and *A. pinodes* strains in lower panel. Chemical structure of ascochitine (upper), solanapyrone A (middle), or pinolidoxin (lower) was shown within each panel.

Metabolic profiles of the 21 strains were obtained using LC-MS analyses of culture extracts. A total of 1,075 unique ions (after data processing, see Methods section) were detected from the extracts. Principal Component Analysis (PCA) was conducted to assess any groupings or trends among the strains. Plots of the PCA scores revealed differences in the metabolic profiles of the known *Ascochyta*/*Phoma* species. The results showed four distinct clusters; *A. pisi* (including *P. koolunga* and the *Ascochyta*-like strains), *A. rabiei*, *P. medicaginis* and *A. pinodes* (together with *A. pinodella*) (Fig. 2). A supervised multivariate analysis using partial least square (PLS) statistics, whereby predetermined groupings by known species were set to classify the dataset, showed that *A. pisi* strains were separated from other strains that were grouped together in the PCA score plot. In the PLS score plot, however, *A. pinodes* and *A. pinodella* strains remained grouped together (Fig. 2).

A hierarchical clustering analysis showed that grouping patterns of strains were consistent with that of PLS analysis (Fig. 2). The *Ascochyta*-like strains (ID1A, ID3A, and G11) were clustered together in the analysis and their metabolic features were distinct from others. Also, *P. koolunga* strain and ID4A strain (isolated from *Lupinus* sp.) were not grouped with *A. pisi* in the hierarchical clustering analysis, but seem to be closer to *A. pisi* than any other species in terms of chemical similarity. The metabolic features of *A. pinodes* and *A. pinodella* were almost identical. Pairwise comparison of total ion chromatograms of representative strains of *A. pisi*, *A. rabiei*, and *A. pinodes* highlighted consistency of the metabolic profile between strains of different geographical origins, as well as the reproducibility of data acquisition (Fig. 2). Also, we detected ascochitine, solanapyrone A, and pinolidoxin, as a major metabolite of *A. pisi*, *A. rabiei*, and *A. pinodes*, respectively (Fig. 2).

### Identification of major secondary metabolites

**Figure 3:**
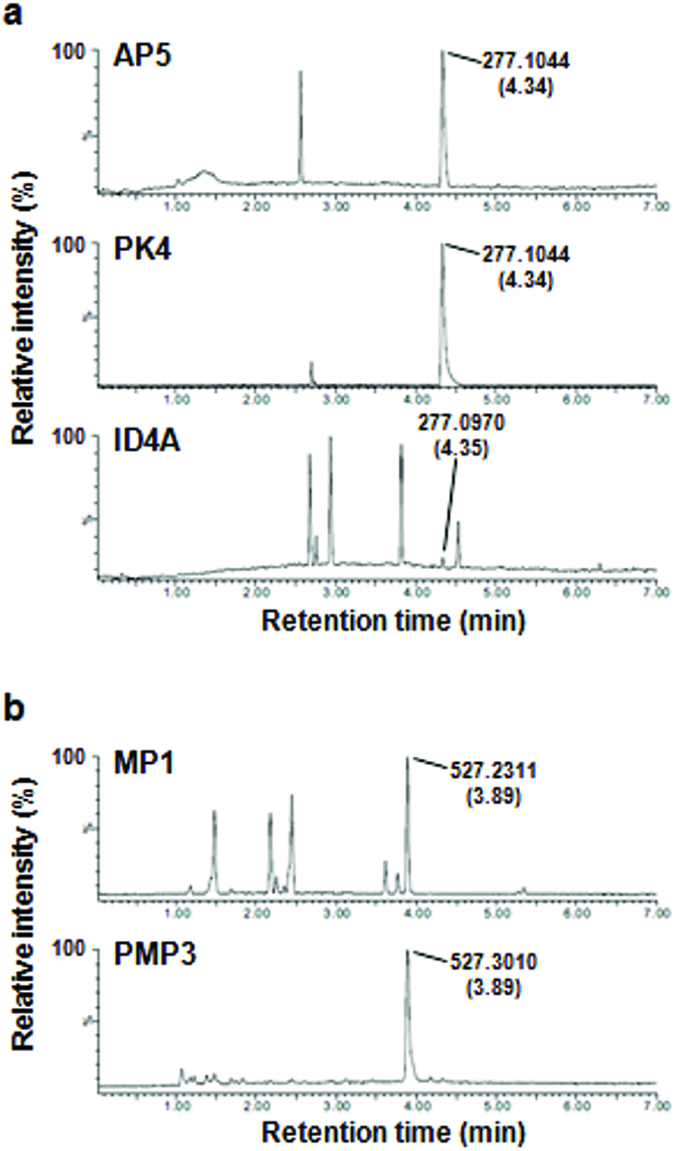
Chromatograms relating to the detection of ascochitine and pinolidoxin. (**a**) Base peak ion chromatograms (BPIs) of AP5, PK4, and ID4A strains. Peaks corresponding to ascochitine were indicated by observed *m/z* values and retention times (in parenthesis). (**b**) BPIs of MP1 and PMP3 strains. Peaks corresponding to pinolidoxin were indicated by observed *m/z* values and retention times (in parenthesis).

PCA and hierarchical clustering analysis showed that *P. koolunga* PK4 strain and ID4A strain were chemically similar to *A. pisi* (Fig. 2). Visual inspection of their chromatograms found that the strains also produce ascochitine (Fig. 3). *A. pinodes* is closely related to *A. pinodella* (syn. *P. medicaginis* var. *pinodella*)^26^,^27^. We also detected pinolidoxin as a major metabolite in all tested *A. pinodella* strains (PMPs) (Fig. 3). Ascochitine and pinolidoxin were purified from the extract of PK4 and PMP3 strains, respectively, and the identities of the purified compounds were confirmed by ^1^H NMR analyses (see Supplementary Fig. S1).

### Chemodiversity

For the second experiment, we analyzed more diverse *Ascochyta*/*Phoma* spp. and ecological strains, including most of the strains used in the first chemometrics analysis. Strains of *A. fabae*, *A. lentis*, and *A*. *viciae-villosae* and strains isolated from different wild vetches (*Vicia* spp.) in Republic of Georgia were included (see Supplementary Table S1). It was previously reported that isolates sampled from wild vetches (strain codes starting with ‘G’) were morphologically indistinguishable from and closely related to *A. fabae*, *A. lentis*, *A. pisi*, and *A*. *viciae-villosae*^26^,^39^. In addition, we analyzed metabolic profiles of strains collected from grasspea (*Lathyrus sativus*) in Italy (strain codes starting with ‘ER’). Also included were *P. herbarum* and *Didymella exigua*, the type species for the genera *Phoma* and *Didymella*.

**Figure 4:**
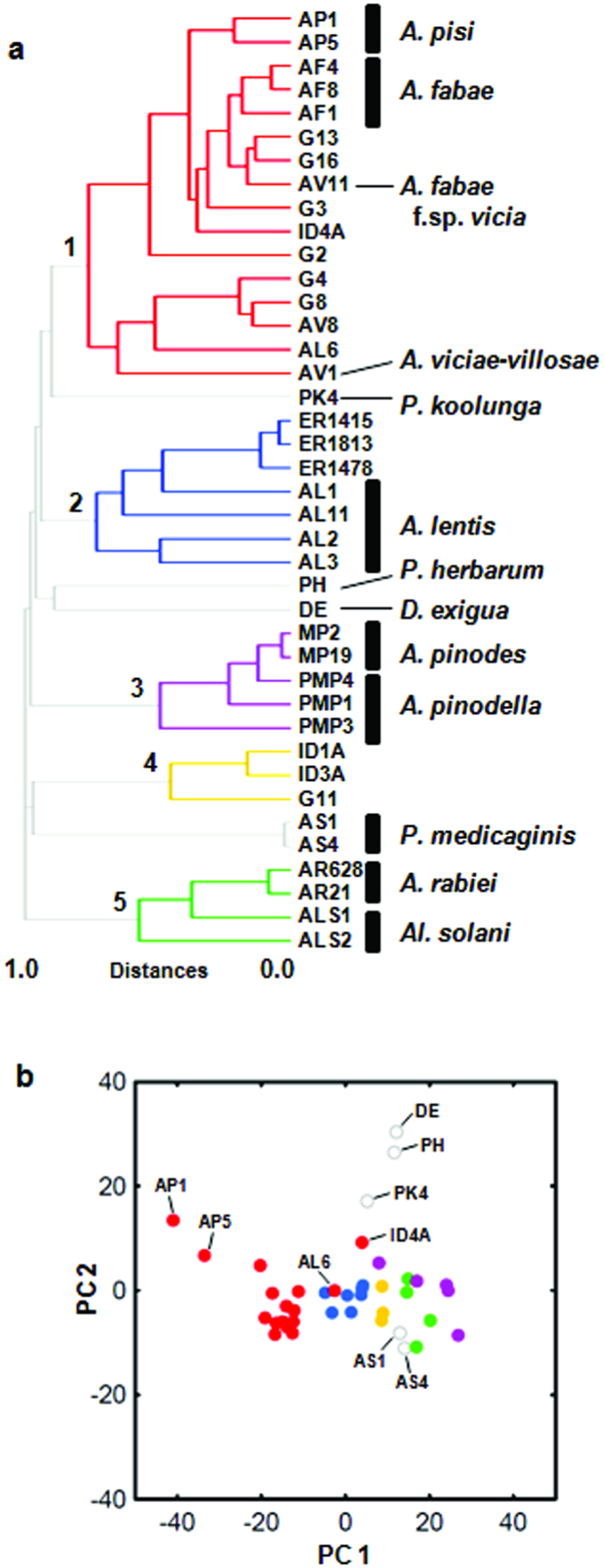
Chemical diversity and grouping observed in 40 *Ascochyta*, *Phoma* and *Alternaria* strains. (**a**) Clustering of fungal strains based on chemical similarity. Numbers and color coding indicate the five main chemical groups according to UPGMA clustering method at a 0.75 distance threshold. (**b**) PLS scores plot, PC1 versus PC 2, showing the supervised separation of the major chemical groups and singletons (grey open circles).

Hierarchical clustering analysis of the LC-MS dataset of the 40 strains detected five major chemically similar groups and strains of *P. koolunga*, *P. medicaginis*, *P. herbarum*, and *D. exigua* as singletons assuming a 0.75 distance threshold (Fig. 4). The ‘chemical group 1′ is comprised of *A. pisi*, *A. fabae*, *A*. *viciae-villosae* and strains isolated from different wild vetches (*Vicia* spp.). Although the metabolic profiles of strains of *A. pisi* and *A. fabae* were highly similar, the two closely related species were still distinguishable by their metabolic features (Fig. 4). The strains isolated from grasspea (ER1415, ER1478, and ER1813) were clustered together with *A. lentis* strains within the ‘chemical group 2′ (Fig. 4), as expected from their phylogenetic similarity. However, the metabolic features of the *A. lentis* strains were highly variable and one strain (AL6) was placed within ‘chemical group 1′.

Consistent with the first analysis, *A. rabiei*, *A. pinodes*/*A. pinodella*, *P. medicaginis*, *P. koolunga*, and the *Ascochyta*-like strains (ID1A, ID3A, and G11) each represented separate chemical groups. In addition, *P. herbarum* (PH) and *Didymella exigua* (DE) strains showed distinct metabolic profiles and were not grouped with any of the legume-associated *Ascochyta*/*Phoma* spp. (Fig. 4). It has been reported that a distantly related plant pathogenic fungus, *Alternaria solani*, produces the same set of SMs, solanapyrones, as does *A. rabiei*^40^. Interestingly, the *Al. solani* strains (ALS1 and ALS2) were grouped together with *A. rabiei* strains, rather than representing an independent group (Fig. 4). The supervised multivariate analysis using PLS statistics also showed a distinction of metabolic features of the five major chemical groups and the singletons (Fig. 4).

### Chemotaxonomy

**Figure 5:**
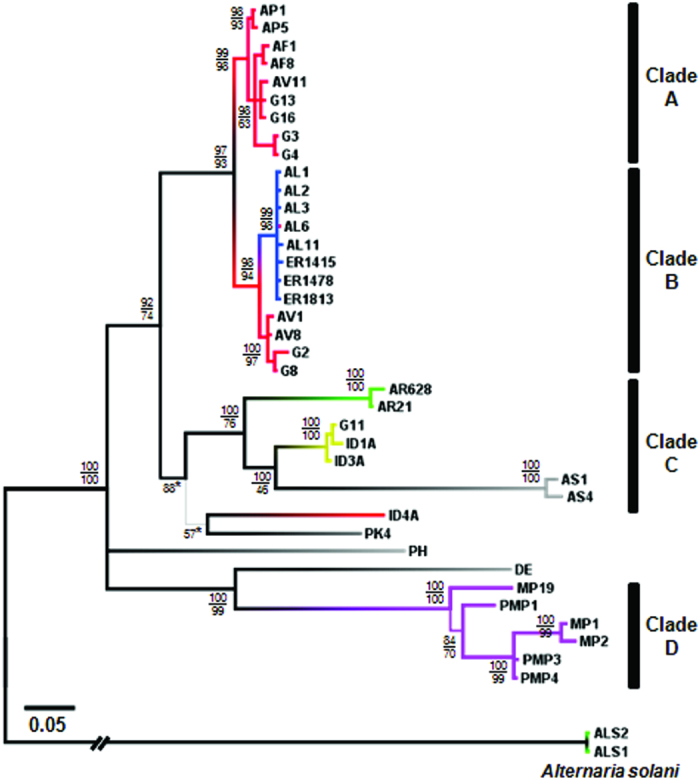
Bayesian phylogeny of *Ascochyta* and *Phoma* species. The phylogeny was estimated from the combined dataset of *CHS*, *EF* and *G3PD* for *Ascochyta* and *Phoma* spp. isolated from various legumes and rooted by *Alternaria solani* strains. The upper numbers at major nodes indicate Bayesian posterior probabilities (PP), and the lower numbers represent bootstrap values (BS) from 1,000 bootstrapped samples in a maximum likelihood (ML) phylogeny. Clades were inferred based on PP greater than or equal to 95% and BS greater than or equal to 70%. (clade A: *A. pisi* [APs] and *A. fabae* [AFs]; clade B: *A. lentis* [ALs] and *A*. *viciae-villosae* [AV1]; clade C: *A. rabiei* [ARs] and *P. medicaginis* [ASs]; clade D: *A. pinodes* [MPs] and *A. pinodella* [PMPs]). The five major chemical groups detected in the hierarchical clustering analysis are mapped on the Bayesian phylogeny, showing distinct metabolic features in evolutionary lineages. Gray lines at the terminal clades indicate ungrouped strains (singletons). Branch lengths are proportional to the inferred amount of evolutionary change and the scale represents 0.05 nucleotide substitutions per site. The asterisks indicate the PP of the corresponding nodes in the Bayesian phylogeny. Placement of these nodes differed under the ML analysis where *P. koolunga* (PK4) and an *Ascochyta*-like strain (ID4A) were placed external to clade C.

To put the observed metabolic profiles in an evolutionary framework, partial DNA sequences of three protein-coding genes, chitin synthase (*CHS*), translation elongation factor alpha (*EF*), and glyceraldehyde 3-phosphate dehydrogenase (*G3PD*) were used to estimate the phylogeny of the strains. The results of both Bayesian phylogeny and Maximum Likelihood are largely congruent (Fig. 5), and the five major chemical groups observed in the hierarchical clustering analysis (Fig. 4) were mapped to the combined phylogeny. The phylogenetic tree revealed four major clades. Within clade A, two subclades corresponding to *A. pisi* and *A. fabae* (including strains isolated from several wild vetches) were evident. Within clade B, two subclades corresponding to *A. lentis* (including strains isolated from grasspea) and *A. viciae-villosae* (including strains isolated from other wild vetches) were evident. Within clade C, three subclades, *A. rabiei*, *P. medicaginis*, and the *Ascochyta*-like strains (ID1A, ID3A, and G11) were evident. Clade D included *A. pinodes* and *A. pinodella*. In addition, we identified putative recombination events as *A. pinodes* strain (MP19) was closer to *A. pinodella* (PMPs) than to other *A. pinodes* strains in the phylogeny, as has been observed previously^26^.

Mapping the five identified chemical groups onto the *Ascochyta*/*Phoma* phylogeny showed that the topology of the chemical phenogram of *Ascochyta*/*Phoma* spp. based on chemical similarity was largely congruent with the topology of the phylogeny estimated from DNA sequence data. In a previous study, strain AV11 isolated from bigflower vetch (*Vicia grandiflora*) received the forma specialis designation (*A. fabae* f. sp. *vicia*)^41^. Given the phylogenetic and chemical distinctiveness (Figs 4 and 5), strains AV11, G13, and G16 appear to be conspecific. Within clade B, the two subclades were chemically distinct (Fig.5). The metabolic features of the *A. lentis* subclade seem to have been diverged from the sister subclade (*A. viciae-villosae*) and clade A (*A. pisi* and *A. fabae*). Within clade C, the metabolic features of each subclade were distinct and appeared to have diversified as these lineages evolved independently. Strains G11, ID1A, and ID3A were phylogenetically and chemically distinct from others and may represent an independent evolutionary lineage. The Bayesian phylogeny showed that *P. koolunga* (PK4) was distinct from other species, so was an unidentified *Ascochyta* sp. (ID4A), but their relative relationship with other species was uncertain (Fig. 5). The lower posterior probability values for clades A, B, and C (92%) were mainly because the two strains were sometimes placed external to the clades containing *A. pisi*, *A. fabae*, *A. lentis*, *A. rabiei* and *P. medicaginis.* The chemotaxonomic data suggested their closer relationship to clades A and B than to clade C, given their chemical similarity to strains in clades A and B and the production of ascochitine in common (Figs 3 and 4).

The phylogenetic tree (Fig. 5) indicated that *A. pinodes*/*A. pinodella*, *P. herbarum* and *D. exigua* are distinct from the other legume-associated *Ascochyta*/*Phoma spp*. (clades A, B, and C). Recently, *A. pinodes* and *A. pinodella* (syn. *P. medicaginis* var. *pinodella*) were transferred to the genus *Peyronellaea* that was elevated from *Phoma* sections^5^. The distinct metabolic features of *A. pinodes*/*A. pinodella* from *P. herbarum*, the type species for the genus *Phoma*, and the other *Ascochyta*/*Phoma* spp. including *P. medicaginis* also justify this reclassification.

## Discussion

Legume-associated *Ascochyta* species are a group of phylogenetically closely related, plant-pathogenic fungi. Due to a paucity of morphological characters, species boundaries within this group have been delimited using phylogenetic and biological species criteria^26^,^27^,^28^,^29^. Ecological divergence based on the evolution of host specificity appears to have played significant roles in the speciation of these legume-associated *Ascochyta* species^26^,^39^. Among host-specific *Ascochyta* fungi, prezygotic isolation barriers are not apparent and therefore some of the recognized species are interfertile producing viable progeny with normal marker segregation^29^. Using whole metabolic profiles as a taxonomic character and its application to chemotaxonomy could aid in the classification of fungal species because distinct metabolic features may reflect lack of gene flow between biologically distinct yet interfertile species. In this study, although potentially recombining taxa (e.g. *A. pinodes* and *A. pinodella*) were chemically indistinguishable, we were able to recognize previously described *Ascochyta* and *Phoma* spp. by their metabolic features. The classification of the legume-associated *Ascochyta*/*Phoma* spp. based on chemical similarity results in a grouping of the strains that is in good agreement with phylogenetic analysis (Fig. 5). Each evolutionary lineage appears to diversify its metabolic capacity and the known *Ascochyta*/*Phoma* spp. exhibited characteristic metabolic profiles.

One of the aims of this study was to resolve evolutionary relationships of ecological strains collected from wild vetches (*Vicia* spp.) and the known *Ascochyta* spp. within clades A and B. Although *A. pisi*, *A. fabae* and *A. fabae* f. sp. *vicia* each exhibited specific metabolic profiles, the phylogenetically well supported subclades including strains from wild vetches did not cluster by chemical similarity. We hypothesize that isolates in clade A may not have chemically diverged due to recent common ancestry. Alternatively, a history of hybridization may have jumbled the metabolic profiles of fungal strains infecting wild plants. In fact, some putative recombination events were detected among strains infecting wild pea (*Pisum elatius*) in *A. pisi* and *A. fabae* clades^26^. Hybridization may benefit fungal species infecting wild plants by modulating a combination of genes related to host specificity, thereby enabling host jump especially when the previous host became no longer available or extinct in wild.

All strains but strain AL6 that belong to the ‘chemical group 1′ produced ascochitine as a major SM (see Supplementary Fig. S2). Ascochitine was initially identified as a selective antifungal agent^42^. Interestingly, ascochitine is widely found in species in genera *Ascochyta* and *Phoma*, including non-legume plant pathogens, *A. hyalospora* and *P. clematidina*^43^,^44^, and a marine-derived fungus *A. salicorniae* (syn. *Stagonosporopsis salicorniae*)^45^. Therefore, ascochitine may be an ancestral SM (a plesiomorphic trait) of legume-associated *Ascochyta*/*Phoma* spp., but has been lost in some lineages in clades B and C.

For clade B, isolates sampled from grasspea (*Lathyrus sativus*) are very similar to *A. lentis* both phylogenetically and chemically and are likely conspecific. However, isolates ER1415 and AL1 sampled from grasspea and lentil, respectively, showed strong host specialization in reciprocal inoculation experiments on their hosts (T.L. Peever, *unpublished*), suggesting that ecological speciation may be underway in these sympatric taxa. We demonstrated that the metabolic profiles of grasspea isolates were highly uniform (Fig. 4). Ascochitine, the signature SM of the ‘chemical group 1′, was not detected in the *A. lentis* subclade (chemical group 2), whose metabolic features were vastly different from those of clade A and the *A. viciae-villosae* subclade B, despite the close phylogenetic relationship. It was recently reported that an *A. lentis* isolate produces anthraquinones and some other SMs^46^, which have not been found in other *Ascochyta* and *Phoma* spp. so far. Interspecific hybrids can be easily generated between *A. lentis* and *A. viciae-villosae* (between the subclades within clade B) under laboratory conditions^29^. However, the clear distinction in metabolic profiles between *A. lentis* and *A. viciae-villosae* suggests that these taxa are genetically isolated in nature and that host specificity has likely prevented hybrid formation and has maintained species integrity^29^.

Metabolic profiles of legume-associated *Ascochyta*/*Phoma* strains were species-specific and consistent among strains of the same species isolated from different geographical regions and host plants, best illustrated by *A. rabiei* from different geographical regions. In the first chemometrics analysis, we included *A. rabiei* strains from cultivated chickpea (*Cicer arietinum*), annual wild chickpea (*C. judaicum*) and perennial wild chickpea (*C*. *montbretti* and *C*. *ervoides*) (see Supplementary Table S1). Significant genetic differentiation was previously reported between *A. rabiei* strains isolated from *C. arietinum* and *C. judaicum*^47^. Nevertheless, the metabolic profiles of AR628 and M305 strains isolated from *C. arietinum* and *C. judaicum* were nearly identical (Fig. 2). This indicated that the metabolic feature is conserved among strains in genetically differentiated populations and can be reproducibly used as a species-specific taxonomic character.

Horizontal gene transfer (HGT) is defined as the movement of stable genetic material between different strains or species^48^. Although rare, several HGT events of SM biosynthesis gene clusters between fungal species have been reported^49^,^50^. It was proposed that the solanapyrone biosynthesis gene cluster in *A. rabiei* was horizontally transferred from *Al. solani* or a distant yet-unknown-species as solanapyrone production is unique to *A. rabiei* among closely related *Ascochyta* spp. and the homologous cluster genes between *A. rabiei* and *Al. solani* share a high degree of DNA sequence similarity (>97%)^51^. Such HGT of SM biosynthesis gene cluster between species may blur species boundaries in chemotaxonomic classification. However, the derived SMs represent autapomorphic traits in a cladistic sense and allow *A. rabiei* to be easily distinguishable from other closely related *Ascochyta* species based on this metabolic feature.

Strains ID1A and ID3A isolated from spotted locoweed (*Astragalus lentiginosus*) in Idaho, USA and strain G11 from tiny vetch (*Vicia hirsuta*) in the Republic of Georgia appear to be conspecific, given the close phylogenetic and chemotaxonomic relationship. The two strains from spotted locoweed appear to be host-specific only causing disease on spotted locoweed but not on other legumes^37^. It remains to be determined if the locoweed strains can cause disease on tiny vetch, and conversely, the tiny vetch strain can cause disease on spotted locoweed. The distinct metabolic feature and DNA sequences may indicate that the strains represent a novel *Ascochyta* or *Phoma* species. In two independent chemometrics analyses, the metabolic features of these isolates always clustered with those of *P. medicaginis* strains (AS1 and AS4), consistent with their close phylogenetic relationship. These two lineages both produce one-celled conidia, while *A. rabiei* strains produce a mixture of one-celled and two-celled conidia, showing an example of the polyphyletic nature of this morphological character.

Based on molecular data, differentiating *A. pinodes* from *A. pinodella* has been difficult^52^,^53^; however, the former is homothallic but the latter is heterothallic^52^,^54^. The metabolic features of these two closely related taxa in clade D were highly similar (Figs 2 and 4). The high level of chemical similarity between these taxa is likely due to sharing a recent common ancestor. However, these taxa also appear to have been affected by more recent gene exchange, possibly via hybrid formation. The putative hybridization or introgression status of strain MP19 needs to be confirmed in future studies. Pinolidoxin is a phytotoxic SM produced by both species^35^. Structurally similar phytotoxic compounds including herbarumin II are also produced by *P. herbarum*^55^. The dienoate group of pinolidoxin at the lactone ring (Fig. 2) can undergo enzymatic hydrolysis, yielding the alcohol derivative, which is herbarumin II. Both pinolidoxin and herbarumin II were found in culture of *A. pinodes*^56^. However, pinolidoxin has not yet been found in *P. herbarum*, suggesting a divergence of the shared biosynthetic pathway in these two evolutionary lineages.

In conclusion, chemotaxonomy in conjunction with phylogenetic analysis may provide novel insights into species delimitation and chemical ecology^57^. The genome would reveal the kinds of metabolites that could potentially be produced in an organism, however, it is the metabolites actually being produced and used during fungal life cycle that are biologically informative and useful for chemotaxonomy. Legume-associated *Ascochyta* and *Phoma* spp. are primarily plant pathogens, but have a significant saprobic phase as with many fungi. The oat kernel cultures supported a uniform growth of the strains as well as consistent production of SMs that were previously described in legume-associated *Ascochyta* and *Phoma* species. The whole metabolome analysis employs simple, fast, and inexpensive growth and extraction methods and relatively short LC-MS analysis time per sample (<15 min), which enables high-throughput chemotaxonomic studies on highly diverse fungal families with minimum available information on their metabolite production.

## Methods

### Fungal strains

Forty-five single-conidial isolates of *Ascochyta* spp., *Phoma* spp., and *Alternaria solani* were obtained from the culture collection maintained by the USDA Western Region Plant Introduction Station, Pullman, Washington. Strains of *A. fabae*, *A. lentis*, *A. pinodella*, *A. pinodes*, *A. pisi*, and *A. rabiei* used in a previous study^26^ were selected for chemotaxonomic analysis. Where possible, we attempted to sample at least 3 representative isolates per species, and we also preferentially selected strains that are deposited in the American Type Culture Collection. Among isolates sampled from various wild vetches (*Vicia* spp.) in the Republic of Georgia in 2004^26^, we selected strains that formed well supported subclades distinct from the above-mentioned *Ascochyta* spp^26^ to compare their metabolic features with those of the known *Ascochyta* spp. Additional isolates used in this study were sampled from *Astragalus lentiginosus*^37^, *Lathyrus sativus*, *Lupinus* sp., and *Medicago sativa*^58^. Six isolates of the recently described species *Phoma koolunga* isolated from *Pisum sativum*^32^ were also included. In a preliminary study, these six isolates had identical metabolic profiles, and the FT07010 strain (recoded as PK4) was chosen for the current study. Finally, we included representative isolates of *P. herbarum* (CBS615.75) and *Didymella exigua* (CBS183.55) as the type species of *Phoma* and *Didymella*, respectively.

### Fungal culture and sample preparation

To obtain the metabolic profiles of fungal strains for chemometrics, all isolates were grown on V8 agar (200 mL V8 juice, 3 g CaCO_3_, 20 g agar in 1 L distilled water) for 10 days. Strains were cultured by inoculating 20 agar plugs (5 mm diameter) into 150-mL Erlenmeyer flasks containing oat kernels (50 mL in volume) that were soaked in water overnight and then autoclaved. The cultures were allowed to grow at 20 °C under an alternating 12 hr photoperiod for 14–18 days until when pycnidial formation indicated by black pigmentation was observed on the oat kernels. Ethyl acetate (EtOAc) was used as a solvent for extraction. To extract metabolites from inoculated oat kernels, 50 mL of EtOAc was added to the culture and gently shaken for 20 minutes at room temperature. Extracts were dried over anhydrous MgSO_4_ and filtered through four layers of cheesecloth to remove oat kernel debris. One and half milliliter of the extracts were transferred to 2-mL screw-cap tubes and centrifuged at 2,200 × g for 5 min. One milliliter of the supernatants were collected and evaporated to dryness on a vacufuge^®^ (Eppendorf, Germany). The same procedure was conducted for autoclaved oat kernels incubated without fungal inoculation to obtain a “blank” extract. Pellets were reconstituted with 200 μL of methanol, and the solutions were subjected to LC-MS analysis.

### Structural identification of ascochitine and pinolidoxin

Ascochitine^33^ and pinolidoxin^35^ were purified from the extracts of *Phoma koolunga* strain PK4 and *Ascochyta pinodella* strain PMP3, respectively. Strains were cultured on 200 g autoclaved oat kernels in a mason jar. After growing the isolates for 2 weeks, oat kernels colonized by either PK4 or PMP3 strain were extracted twice with 150 mL of EtOAc. The combined extracts were dried over MgSO_4_ and filtered through four layers of cheesecloth to remove oat kernel debris and MgSO_4_. The filtrate was concentrated by a rotary evaporator. Ascochitine was semi-purified by following an acid-base extraction procedure described in literature^59^. The resulting residue was further purified by preparative TLC on silica gel using a mixture of dichloromethane/methanol/formic acid (96:3:1) to afford ascochitine as yellow oil (2.6 mg). For pinolidoxin purification, flash column chromatography was performed using a mixture of EtOAc/hexanes (2:3) on a silica gel to give a white solid (5.8 mg). Fractions containing pinolidoxin were recognized by its known mass spectra^56^. The purified compounds were further identified by performing ^1^H NMR analyses (see Supplementary Fig. S1). The spectral characterization of pinolidoxin was compared with literature ^1^H NMR data^60^.

### Mass spectrometric analyses

Chromatographic separation was achieved using an ACQUITY UPLC system (Waters Corp., USA) as described elsewhere^51^. MS analysis was performed on an inline Synapt G2-S HDMS (Waters Corp.) time of flight mass spectrometer. For positive mode, the electrospray ionization (ESI) conditions were as follows: capillary voltage 3.65 kV; source temperature 120 °C; cone voltage 30 V; desolvation temperature 250 °C; desolvation gas flow 800 L h^−1^ (N_2_ gas); collision gas flow 2.0 mL min^−1^ (argon gas); data acquisition range *m/z* 50–1,000. For negative mode, the ESI conditions were as follows: capillary voltage 2.5 kV; source temperature 90 °C; cone voltage 40 V; desolvation temperature 150 °C; desolvation gas flow 500 L h^−1^ (N_2_ gas); collision gas flow 2.0 mL min^−1^ (argon gas); data acquisition range *m/z* 50–1,000. Leucine encephalin was used as the lock mass (*m/z* 556.2771 in ESI^+^ and 554.2615 in ESI^−^) at a concentration of 200 ng mL^−1^ and flow rate of 10 μL min^−1^, with a lockspray frequency of 30 s. For the TOF experiments, data were acquired in the MS^E^ mode in which two separate scan functions were programmed for the MS acquisition method. One scan function was set at low collision energy (trap at 4 eV and transfer at 2 eV), and the other scan function was set at high collision energy (trap ramped from 15–50 eV and transfer at 2 eV). The mass spectrometer switched rapidly between the two functions during data acquisition. As a result, information on intact precursor ions and on product ions was obtained from a single LC run.

### Chemometrics analyses

We performed two independent chemometrics analyses using LC-MS datasets. In the first experiment, the LC-MS dataset of fungal extracts of 21 strains was acquired in positive mode. In the second experiment, to reproduce the first experiment and analyze more diverse taxa, the LC-MS dataset of fungal extracts of 40 strains was acquired with both positive and negative modes. The LC-MS datasets were further processed to extract and align peaks from the chromatograms of strains, using Progenesis QI software (Waters Corp.). To discard peaks corresponding to extremely polar and nonpolar compounds as well as noise peaks, the early- and late-eluting peaks (<0.5 min and >7.0 min in the chromatograms) and the peaks with absolute ion intensity less than 100 were excluded from the analyses, resulting in a total of 1,733 and 4,610 putative metabolic features (variables) for the first and second datasets, respectively. To aid the metabolic profiling process, the LC-MS data of the “blank” extract (oat kernel only) for each dataset were also analyzed to extract metabolic features and to use as a background reference. These reference ion peaks were removed from all fungal extracts data in the matrices, resulting in a total of 1,075 and 2,770.

The resulting data matrices (21 observations with 1,075 variables and 40 observations with 2,770 variables; see Supplementary Dataset 1) were exported as comma-separated value files and imported to Statistica v.12 (StatSoft, Inc., USA). All data were normalized prior to multivariate analysis, using the statistical method implemented in Progenesis QI (Waters Corp.). Principal component analysis (PCA) was initially performed to assess any grouping or trends among *A. rabiei*, *A. pisi*, and *A. pinodes* strains in the first dataset, using the Statistica software (StatSoft, Inc., USA). To achieve better clustering of data points, supervised partial least square (PLS) analyses were performed, in which predetermined groupings by known species were set to classify the datasets. For hierarchical clustering analyses, matrices of Pearson correlation coefficients for the datasets were constructed using the MultiExperiment Viewer (MeV) software (Institute for Genomic Research, CA, USA). The robustness of the clusters produced was determined by looking at the consensus of the two clustering methods, complete linkage and unweighted pair group method with arithmetic mean (UPGMA). Both methods agreed in dividing strains into five main clusters and several singletons in the second dataset.

### DNA sequencing

For phylogenetic analyses, genomic DNA was extracted from lyophilized mycelium as described previously^26^, and partial DNA sequences of three protein-coding genes (*CHS*, *EF*, and *G3PD*) were used^26^. To obtain the DNA sequences from the strains, primers (CHS-79 and CHS-354 for *CHS* gene; EF1-728F and EF1-986R for *EF* gene; gpd-1 and gpd-2 for *G3PD* gene) were used for PCR amplification^61^,^62^. PCR condition consisted of 95 ºC for 3 min followed by 32 cycles of 95 °C for 30 s, 52 °C for 30 s, and 72 °C for 45 s. Amplicons were direct sequenced for each strand and the sequences were deposited under GenBank accession numbers KR184153–KR184188. Additional DNA sequences used in previous phylogenetic studies^26^ were also retrieved from GenBank.

### Phylogenetic analysis

A phylogeny was estimated from the combined *CHS*, *EF* and *G3PD* dataset in a Bayesian framework using Markov chain Monte Carlo (MCMC) sampling in MrBayes version 3.2^63^. The sequence alignment length was 1,246-nucleotide(nt)-long (*CHS*: 330 nt; *EF*: 366 nt; *G3PD*: 550 nt), and the concatenated dataset were deposited in TreeBASE (Study 17,485, www.treebase.org). A partitioned analysis was implemented in MrBayes with evolutionary models and parameters for base frequency, substitution rates, and gamma distribution shape, which were estimated independently for each gene by Peever *et al.*^26^. Each run of the sampler consisted of 2,000,000 generations of the Markov chain. Four chains were run in each analysis (one heated and three cold) with the temperature parameter set at 0.1 and random chains swapped one time per generation. Two independent analyses (each of 2,000,000 generations) each were started from a random tree. Trees were sampled every 500 generations and the first 500,000 generations (1,000 trees) of each analysis were discarded as burn-in. Average posterior probabilities (PP) were estimated for each node of the phylogeny across runs (6,000 trees from 2,000,000 total generations of the MCMC). The phylogeny was rooted by *Alternaria solani*. A maximum likelihood (ML) phylogeny based on the general time reversible model was constructed using MEGA6 software^64^. A discrete gamma distribution with invariant sites was used to model evolutionary rate differences among sites. For the heuristic search of initial trees, Neighbor-Join and BioNJ algorithms were applied as a default. Nodal support was evaluated using 1,000 bootstrapped datasets. Clades were inferred based on PP greater than or equal to 95% and BS greater than or equal to 70%.

## Additional Information

**How to cite this article**: Kim, W. *et al.* Use of metabolomics for the chemotaxonomy of legume-associated *Ascochyta* and allied genera. *Sci. Rep.* **6**, 20192; doi: 10.1038/srep20192 (2016).

## Supplementary Material

Supplementary Information

Supplementary Dataset 1
