# Supplementary material for: Use of metabolomics for the chemotaxonomy of legume-associated Ascochyta and allied genera
Source: Sci Rep. 2016 Feb 5;6:20192. doi: 10.1038/srep20192 (PMC4742866; doi:10.1038/srep20192)
Supplement: Supplementary Information [file srep20192-s1.pdf]

Supplementary information

Use of metabolomics for the chemotaxonomy of legume-associated *Ascochyta* and allied genera

Wonyong Kim, Tobin L. Peever, Jeong-Jin Park, Chung-Min Park, David R. Gang, Ming Xian, Jenny A. Davidson, Alessandro Infantino, Walter J. Kaiser and Weidong Chen

Supplemental Table S1. List of *Ascochyta*, *Phoma* and *Alternaria* strains used in this study

Fungal species	Strain code[a]	Host plant	Location	Collector	Year	Reference	GenBank accession numbers[b]		
							G3PD	*CHS*	*EF*
Ascochyta rabiei	AR628 (201622)[cd]	*Cicer arietinum*	Malikiye, Syria	J. Geistlinger	1995	Peever et al. (2007)	DQ383958	KR184160	KR184176
A. rabiei	AR21 (76502)[cd]	*C. arietinum*	Idaho.USA	W.J. Kaiser	1996	Peever et al. (2007)	DQ383958	DQ386480	DQ386488
A. rabiei	AR738 (201625)[c]	*C. montbretti*	Bulgaria	W.J. Kaiser	1996	Peever et al. (2007)	–	–	DQ386490
A. rabiei	Georgia-10[c]	*C. ervoides*	Ateni, Georgia	W.J. Kaiser	2004	Peever et al. (2007)	–	–	DQ386491
A. rabiei	M305[c]	*C. judaicum*	Ramot Menashe, Israel	O. Frenkel	2005	Frenkel et al. (2010)	–	–	–
A. lentis	AL1 (96419)[d]	*Lens culinaris*	Australia	W.J. Kaiser	–	Peever et al. (2007)	DQ383961	DQ386482	DQ386493
A. lentis	AL2 (96420)[d]	*L. culinaris*	Brazil	W.J. Kaiser	–	Peever et al. (2007)	DQ383961	DQ386482	DQ386493
A. lentis	AL3 (46979)[d]	*L. culinaris*	Canada	W.J. Kaiser	–	Peever et al. (2007)	DQ383961	DQ386482	KR184174
A. lentis	AL6 (46982)[d]	*L. culinaris*	Russia	W.J. Kaiser	–	Peever et al. (2007)	DQ383961	DQ386482	DQ386493
A. lentis	AL11 (46980)[d]	*L. culinaris*	India	W.J. Kaiser	–	Peever et al. (2007)	DQ383962	DQ386482	DQ386493
A. pisi	AP1 (201617)[cd]	*Pisum sativum*	Bulgaria	W.J. Kaiser	1992	Peever et al. (2007)	DQ383963	DQ386481	DQ386494
A. pisi	AP4 (210620)[c]	*P. sativum*	Saskatoon, Canada	B. Gossen	–	Peever et al. (2007)	DQ383964	–	–
A. pisi	AP5[cd]	*P. sativum*	Saskatoon, Canada	B. Gossen	–	Peever et al. (2007)	DQ383965	DQ386481	DQ386494
A. fabae	AF1 (96418)[d]	*Vicia faba*	Saskatoon, Canada	B. Vandenberg	1992	Peever et al. (2007)	DQ383959	DQ386481	DQ386492
A. fabae	AF4 (96409)	*V. faba*	Iran	C. Bernier	1992	Peever et al. (2007)	–	–	–
A. fabae	AF8 (201613)[d]	*V. faba*	Cambridge, UK	R.A.A. Morrall	1992	Peever et al. (2007)	DQ383960	DQ386481	DQ386492
A. pinodes	MP1 (201628)[cd]	*P. sativum*	Oregon, USA	J. Baggett	–	Peever et al. (2007)	DQ383975	KR184166	KR184181
A. pinodes	MP2 (201629)[cd]	*P. sativum*	Ireland	J. Kraft	–	Peever et al. (2007)	DQ383976	KR184167	KR184181
A. pinodes	MP19 (201632)[cd]	*P. sativum*	Argentina	D. Webster	1996	Peever et al. (2007)	DQ383977	KR184168	KR184182
A. pinodella	PMP1[cd]	*L. culinaris*	–	W.J. Kaiser	–	Chilvers et al. (2009)	DQ383978	KR184169	KR184183
A. pinodella	PMP3 (58660)[cd]	*L. culinaris*	Washington, USA	W.J. Kaiser	1996	Peever et al. (2007)	DQ383978	KR184170	KR184184
A. pinodella	PMP4 (58662)[cd]	*C. arietinum*	Washington, USA	W.J. Kaiser	1996	Peever et al. (2007)	DQ383979	KR184170	KR184185
A. viciae-villosae.	AV1[d]	*V. villosa*	Washington, USA	W.J. Kaiser	1983	Peever et al. (2007)	DQ383968	DQ386483	DQ386497
Ascochyta sp.	AV8[d]	*V. lathyroides*	Washington, USA	W.J. Kaiser	1994	Peever et al. (2007)	DQ383969	DQ386483	DQ386497
Ascochyta sp.	AV11[d]	*V. grandiflora*	Connecticut, USA	K.T. Leath	1994	Peever et al. (2007)	DQ383970	DQ386484	DQ386498

Table S1. (Continued)

Fungal species	Strain code[a]	Host plant	Location	Collector	Year	Reference	GenBank accession numbers[b]		
							G3PD	*CHS*	*EF*
Ascochyta sp.	Georgia-2[d]	*Vicia grandiflora*	Dedoplistskaro, Georgia	W.J. Kaiser	2004	Peever et al. (2007)	DQ383971	DQ386485	DQ386499
Ascochyta sp.	Georgia-3[d]	*V. grandiflora*	Dedoplistskaro, Georgia	W.J. Kaiser	2004	Peever et al. (2007)	DQ383972	DQ386486	DQ386500
Ascochyta sp.	Georgia-4[d]	*V. sepium*	Manglesi, Georgia	W.J. Kaiser	2004	Peever et al. (2007)	DQ383972	DQ386486	DQ386501
Ascochyta sp.	Georgia-8[d]	*V. grandiflora*	Ateni, Georgia	W.J. Kaiser	2004	Peever et al. (2007)	DQ383971	DQ386483	DQ386502
Ascochyta sp.	Georgia-13[d]	*V. grandiflora*	Ateni, Georgia	W.J. Kaiser	2004	Peever et al. (2007)	DQ383970	DQ386481	DQ386505
Ascochyta sp.	Georgia-16[d]	*V. cordata*	Tserovani, Georgia	W.J. Kaiser	2004	Peever et al. (2007)	DQ383974	DQ386481	DQ386506
Ascochyta sp.	ER1415[d]	*Lathyrus sativus*	Salerno, Italy	A. Infantino	2007	–	DQ383961	DQ386482	KR184175
Ascochyta sp.	ER1478[d]	*La. sativus*	Salerno, Italy	A. Infantino	2008	–	DQ383961	DQ386482	DQ386493
Ascochyta sp.	ER1813[d]	*La. sativus*	Salerno, Italy	A. Infantino	2013	–	DQ383961	DQ386482	DQ386493
Ascochyta sp.	Georgia-11[cd]	*V. hirsuta*	Ateni, Georgia	W.J. Kaiser	2004	Chilvers et al. (2006)	DQ383973	DQ386487	DQ386504
Ascochyta sp.	ID1A[cd]	*Astragalus* sp.	Idaho, USA	W.J. Kaiser	2005	Habibi et al. (2015)	KR184154	KR184161	KR184177
Ascochyta sp.	ID3A[cd]	*Astragalus* sp.	Idaho, USA	W.J. Kaiser	2005	Chilvers et al. (2009)	KR184155	DQ386487	KR184178
Ascochyta sp.	ID4A[cd]	*Lupinus* sp.	Idaho, USA	W.J. Kaiser	2005	Chilvers et al. (2009)	KR184156	KR184162	KR184179
Phoma medicaginis	AS1[cd]	*Medicago sativa*	Washington, USA	T.L. Peever	2001	Chilvers et al. (2009)	EU394712	KR184163	EU394714
P. medicaginis	AS4[cd]	*M. sativa*	Washington, USA	T.L. Peever	2001	Akamatsu et al. (2008)	EU394713	KR184164	EU394715
P. koolunga	PK4 (FT07010)[cd]	*Pisum sativum*	Australia	J.A. Davidson	2007	Davidson et al. (2009)	KR184157	KR184165	KR184180
P. herbarum	PH (CBS615.75)[d]	*Rosa multiflora*	Netherlands	G.H. Boerema	1973	Chilvers et al. (2009)	KR184158	KR184171	KR184186
Didymella exigua	DE (CBS183.55)[d]	*Rumex arifolius*	France	E. Müller	1953	Chilvers et al. (2009)	KR184159	KR184172	KR184187
Alternaria solani	ALS1[d]	*Solanum*	Washington, USA	D.A. Johnson	2011	Kim et al. (2015)	KR184153	KR184173	KR184188
Al. solani	ALS2[d]	*tuberosum*	Idaho, USA	D.A. Johnson	2010	Kim et al. (2015)	KR184153	KR184173	KR184188

[a] American Type Culture Collection accession numbers (or CBS codes for *Phoma herbarum* and *Didymella exigua*) in parentheses, where applicable, and the strain codes for Georgia-XX strains were shortened as G-XX in the text.

[b] GenBank accessions numbers for glyceraldehyde 3-phosphate dehydrogenase (*G3PD*), chitin synthase (*CHS*), and translation elongation factor alpha (*EF*).

[c] Strains used in the first chemometrics analysis. [d] Strains used in the second chemometrics analysis.

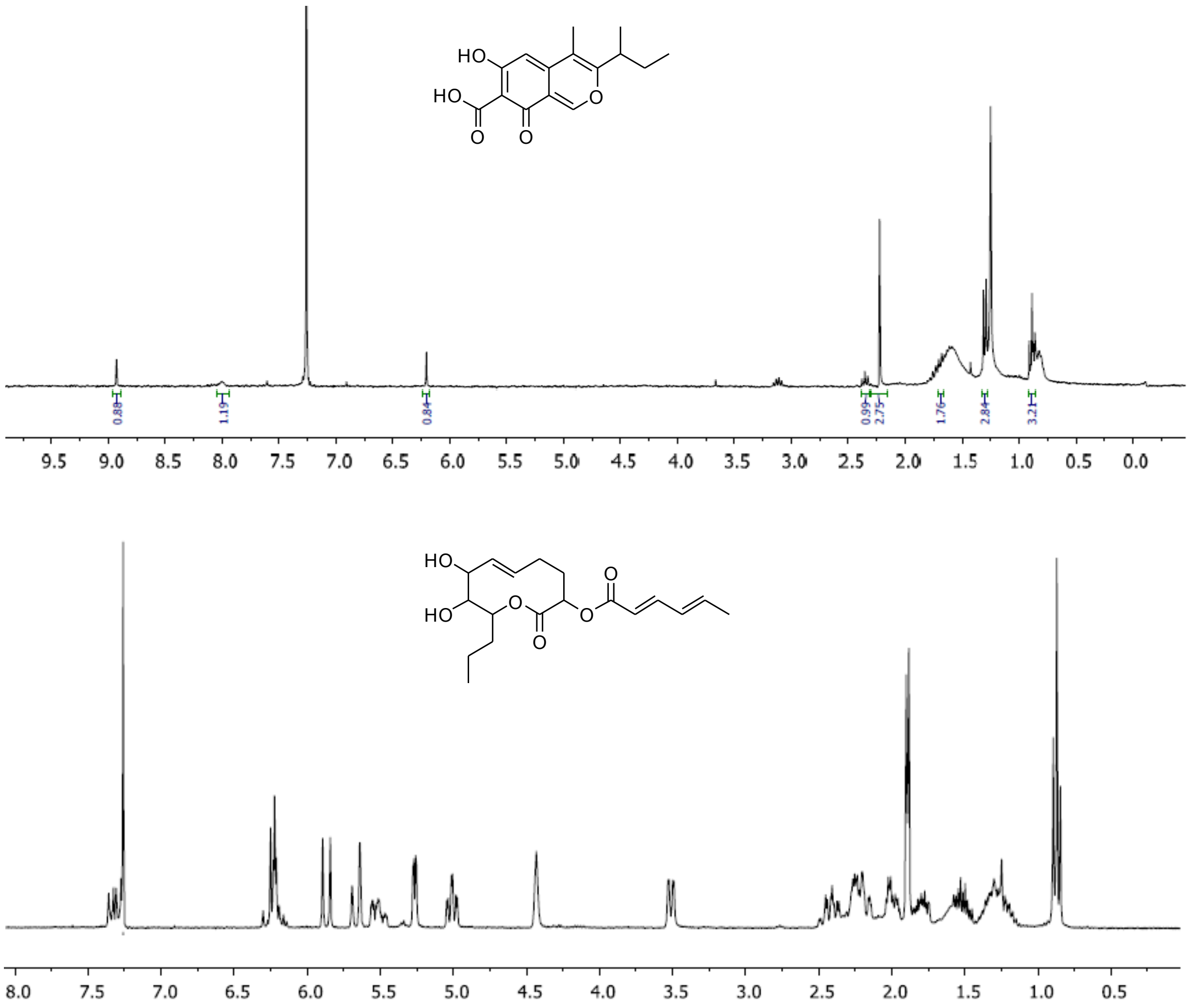

Figure S1. Structural identification of ascochitine and pinolidoxin. (a) 300 MHz ^{1}H NMR in CDCl$_3$ (ppm) for ascochitine purified from culture of *Phoma koolunga* (strain PK4). (b) 300 MHz ^{1}H NMR in CDCl$_3$ (ppm) for pinolidoxin purified from culture of *Ascochyta pinodella* (strain PMP3).

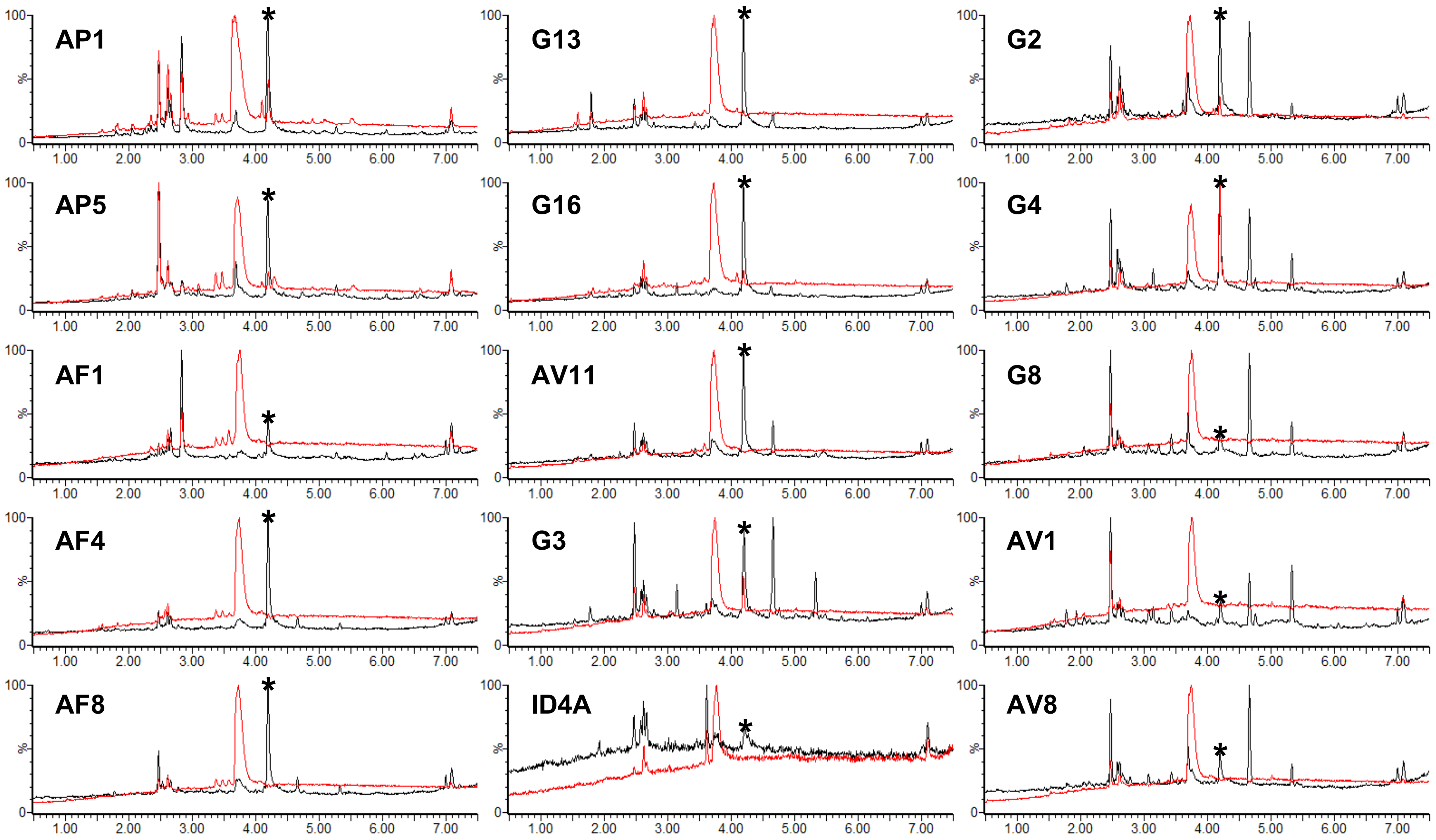

Figure S2. Overlaid total ion chromatograms of strains belonging to ‘chemical group 1’ in positive mode (black lines) and negative mode (red lines). Peaks corresponding to ascochitine were indicated by asterisks and identified by comparing their mass spectra and retention times with that of ascochitine reference. x-axis; retention time (min), y-axis; relative ion intensity (%).
